# A lightweight Deeplab V3+ network integrating deep transitive transfer learning and attention mechanism for burned area identification

**DOI:** 10.1038/s41598-024-66060-7

**Published:** 2025-05-07

**Authors:** Lizhi Liu, Ying Guo, Erxue Chen, Zengyuan Li, Yu Li, Yang Liu, Qiang Zhang, Bing Wang

**Affiliations:** 1 College of Horticulture and Forestry, Tarim University, Alar, Xinjiang, 843300 China; 2 Institute of Forest Resource Information Techniques, Chinese Academy of Forestry, BeiJing, 100091 China; 3 Key Laboratory of Forestry Remote Sensing and Information System of National Forestry and Grassland Administration, Chinese Academy of Forestry, Beijing, 100091 China; 4 School of Geomatics, Liaoning Technical University, Fuxin, 123000 China; 5 College of Forestry, Inner Mongolia Agricultural University, Hohhot, China

**Keywords:** Forestry, Computer science

## Abstract

Complete and accurate burned area map data are needed to document spatial and temporal patterns of fires, to quantify their drivers, and to assess the impacts on human and natural systems. To achieve the the purpose of identifying burned area accurately and efficiency from remote sensing images, a lightweight deep learning model is proposed based on Deeplab V3 + , which employs the combination of attention mechanism and deep transitive transfer learning (DTTL) strategy. The lightweight MobileNet V2 network integrated with Convolutional Block Attention Module (CBAM) is designed as the backbone network to replace the traditional time-consuming Xception of Deeplab V3 +. The attention mechanism is introduced to enhance the recognition ability of the proposed deep learning model, and the deep transitive transfer learning strategy is adopted to solve the problem of incorrect identification of the burned area and discontinuous edge details caused by insufficient sample size during the extraction process. For the process of DTTL, the improved Deeplab V3 + network was first pre-trained on ImageNet. Sequentially, WorldView-2 and the Sentinel-2 dataset were employed to train the proposed network based on the ImageNet pre-trained weights. Experiments were conducted to extract burned area from remote sensing images based on the trained model, and the results show that the proposed methodology can improve extraction accuracy with OA of 92.97% and Kappa of 0.819, which is higher than the comparative methods, and it can reduce the training time at the same time. We applied this methodology to identify the burned area in Western Attica region of Greece, and a satisfactory result was achieved with. OA of 93.58% and Kappa of 0.8265. This study demonstrates the effectiveness of the improved Deeplab V3 +in identifying forest burned area. which can provide valuable information for forest protection and monitoring.

## Introduction

Forest fires represent a significant natural disaster, exerting profound impacts on the local ecosystem. Following a fire, the forest’s ecosystem sustains severe damage, profoundly altering soil chemistry, surface runoff patterns, surface heat, and energy balance^1^. Large-scale forest fires not only result in substantial carbon emissions but also have far-reaching consequences on local and even global climates. Identifying burned areas is paramount in estimating the extent of fire damage and assessing its severity^2^.

Currently, there are two commonly used methods for extracting burned area. Although field measurement, has high extraction accuracy, it is labor-intensive and expensive, especially for the large-scale burned area. As an excellent earth observation technology, remote sensing data have been widely employed in the field of target identification tasks, with the advantages of low cost and fast speed. In the field of using remote sensing images to extract burn areas, research can be divided into two main categories: index methods and machine learning methods. The index method has evolved over time, starting with the widely used Normalized Difference Vegetation Index (NDVI) and progressing to the Global Environment Monitoring Index (GEMI)^3^. Subsequently, the Burned Area Index (BAI)^4^ and the Normalized Burn Ratio (NBR^5,6^) were introduced. Although the index method can quickly extract burned area, it is inevitable that the index method will cause a large number of misclassifications. Therefore, it is necessary to use methods such as machine learning to improve accuracy on the basis of calculating the index^7,8^. (2) Machine learning method: In the traditional machine learning algorithm, commonly used machine learning methods include support vector machines, random forests, decision trees, k-nearest neighbors, etc.^9–11^. Burned area extraction is also widely used. Among them^12^, used the C-means clustering method to calculate the mid-infrared burn index Mid-Infrared Burn Index (MIRBI) data. The Overall Accuracy reaches more than 90%. As a result, machine learning can improve the precision and accuracy to a certain extent when extracting burned area. However, this type of method has high requirements on the quality of training data and feature selection, which makes it difficult to extract on a global scale. In today ‘s machine learning algorithms, deep learning methods are widely used. For deep learning methods to extract burned area, currently commonly used methods mainly include Convolutional Neural Network (CNN), Recurrent Neural Network (RNN) and Generative Adversarial Network (GAN)^13–15^. The application of deep learning networks for burned area identification can be applied globally and has the advantages of high accuracy and strong adaptability. However, most of the existing burned area extraction networks rely on extremely large prior training samples and training takes a long time.

In recent years, the rapid development of fully convolutional neural networks has accelerated the advancement of remote sensing image classification technology^16^. Building upon this foundation, many scholars have proposed optimized network models to address various land feature classification needs by improving network structures. For example, in the research on the U-Net network, Shi et al.^17^ continuously adjusted U-Net and RefineNet networks based on the idea of adversarial learning, and applied them to extract features of urban villages. Compared to the original network structure, this method can effectively extract features of urban villages and significantly improve extraction accuracy. He et al.^18^ fused dilated convolutions into the U-Net network to extract buildings in high-resolution remote sensing images. With the different convolution rates in dilated convolutions, this network can recognize buildings of different sizes, achieving significant improvements in both accuracy and performance over the original network. Additionally, Li et al.^19^ fused multi-head attention into the U-Net network. This algorithm has the advantages of a small model size and few parameters, and achieves good classification results in various public datasets for remote sensing image classification tasks. In the research on the SegNet network^20^, selected appropriate convolutional layer numbers and applied transfer learning to address the problem of low classification accuracy in remote sensing image classification with limited sample datasets. Furthermore, Zhang et al.^21^ fused pooling indices and convolution to incorporate semantic information into the SegNet network. This method improves the accuracy of land feature classification in complex rural scenes compared to the original network. Meanwhile, compared to U-Net and SegNet networks, the Deeplab v3 network integrates spatial pyramid pooling modules, enabling multi-scale recognition and achieving higher accuracy, thus attracting significant attention. Han et al.^22^ addressed the road extraction problem in remote sensing images by improving the Deeplab v3 network. Compared with other road extraction methods, this algorithm demonstrates higher extraction accuracy and completeness. Additionally, Xu et al.^23^ fused adaptive loss functions into the Deeplab v3 +network, enabling accurate and rapid extraction of buildings in high-resolution remote sensing images.

The training of deep neural networks demands a substantial amount of labeled sample images, making sample creation a complex task. Particularly in remote sensing image extraction tasks, achieving high-precision classification with fewer labeled samples and limited iterations poses a significant challenge. Transfer learning, as highlighted by Pan and Yang^24^, offers a solution by leveraging pre-existing knowledge from large datasets like ImageNet. Models trained on ImageNet exhibit rich underlying features, making fine-tuning with ImageNet pre-trained models a prevalent deep transfer learning method. For instance, Li et al.^33^ utilized the Inception V3 network pre-loaded with ImageNet weights to extract features from remote sensing images. By training a single-layer fully connected layer with a small amount of labeled remote sensing data, they achieved high classification accuracy in remote sensing image scene recognition. Similarly, Teng et al. (2020) employed a superpixel segmentation algorithm to classify forestland images. They fine-tuned a network with ImageNet pre-trained weights, achieving higher classification accuracy and more precise tree species boundaries. While ImageNet pre-trained weights can partly leverage its rich features, there’s often a significant disparity between ImageNet image features and those of remote sensing images, limiting the effectiveness of remote sensing target extraction. Additionally, the issue of lengthy training times can be addressed by employing lightweight networks, although this approach might sacrifice extraction accuracy.

To tackle these challenges, this paper proposes a burned area extraction method for remote sensing images based on deep transfer learning, integrating the concept of transitive transfer learning with deep neural networks. This method enhances the alignment between ImageNet and remote sensing image burned area datasets by incorporating an intermediate domain dataset derived from open-source remote sensing scene recognition. Furthermore, it enhances traditional deep neural networks by designing the Deeplab V3 +network, with an improved MobileNet V2 as the backbone. This design enhances the effectiveness of transfer learning while retaining the rich underlying features of ImageNet weights. The proposed method addresses the practical task of extracting burned areas from remote sensing images. With limited annotated samples, it enhances the accuracy of burned area extraction and enables precise and rapid extraction.

## Methods

This article makes three improvements based on the original Deeplab V3 +network. (1) The MobileNet V2 network is used as a replacement for the Xception network in the backbone network of the Deeplab V3 +network to address the issue of long training time. (2) The CBAM is referenced in the MobileNet V2 backbone network to improve the network’s capability to identify forest burned area. (3) To tackle the problem of reduced accuracy in burned area caused by insufficient sample size, the improved Deeplab V3 + network incorporates the deep transitive transfer learning strategy.

**Figure 1:**
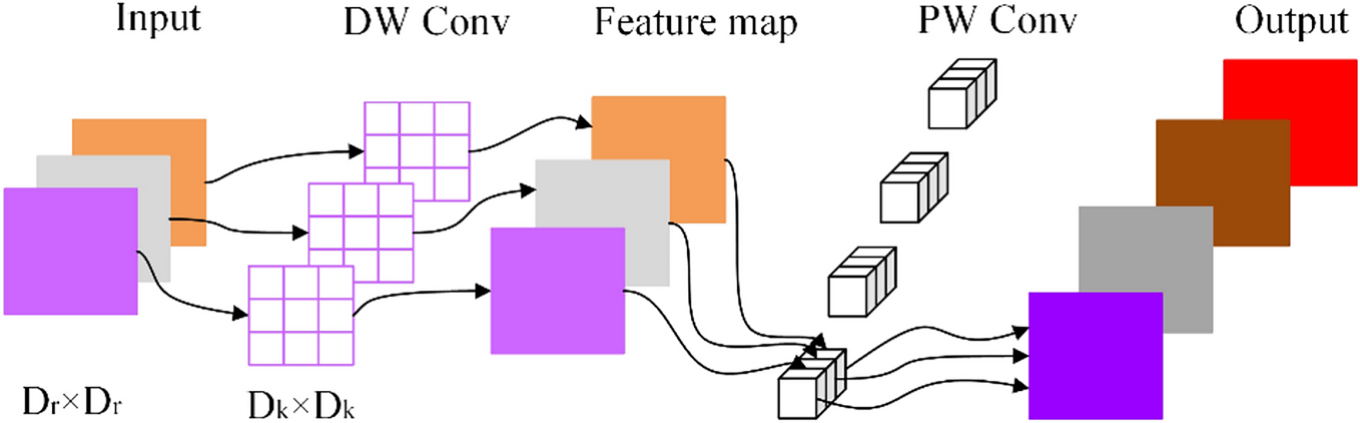
Depthwise separable convolution.

*MobileNet V2 network* MobileNet V2 is a lightweight network model proposed by the Google team in 2016 (Sandler et al., 2020), which continues the depth-separable convolution idea utilized in the MobileNet V1 network (Howard et al., 2017). The basic process of depthwise separable convolution is shown in Fig. 1. In the DepthWise Convolution (DW) step, each channel of the input is convolved with an independent convolution kernel. This means that for each channel in the input, there is a corresponding convolution kernel performing convolution operation. Such operation preserves the depth information of the input and allows the model to learn features for each channel. In the PointWise Convolution (PW) step, typically a 1 × 1 convolution kernel is used to convolve the output of the Depthwise Convolution. The purpose of this step is to linearly combine the output of the depthwise convolution to generate the final output feature map. Pointwise Convolution can be seen as combining and adjusting features at each position to obtain a richer feature representation. Overall, depth separable convolution reduces the number of parameters and computational cost by separating convolution operations across spatial and channel dimensions, while still maintaining good feature representation capability. Hence, it has been widely used in many lightweight and real-time applications. Compared with ordinary convolution, depth-separable convolution combined with DepthWise Convolution and PointWise Convolution can effectively reduce model parameters. In addition, the inverted residual structure and linear bottleneck structure are introduced on the basis of the MobileNet v1 network, which significantly speeds up the convergence of the model and further reduces the calculation amount.

**Figure 2:**
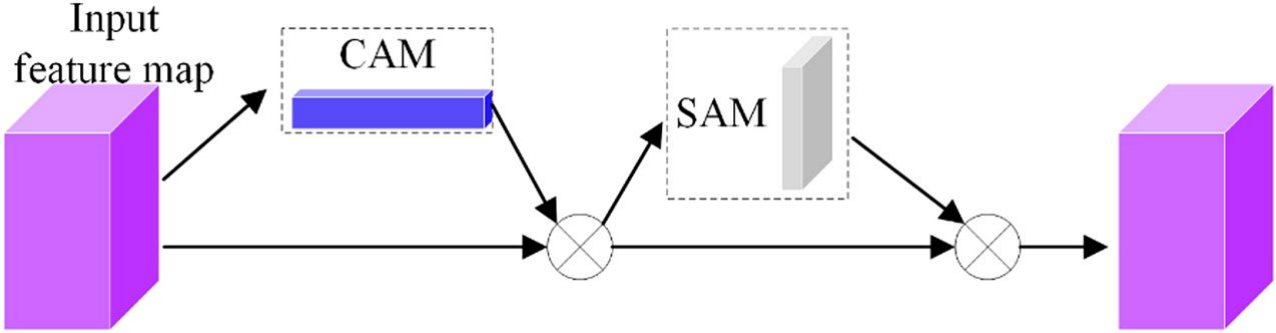
CBAM module.

*Attention mechanism* Convolutional Block Attention Module (CBAM), as an attention mechanism, can effectively enhance the expressive ability of convolutional neural networks. It is composed of Channel Attention Mechanism (CAM)^29–31^ and Spatial Attention Mechanism (SAM)^32–34^ connections and is a lightweight attention module. The CBAM module sequentially processes feature maps from two different dimensions: channel and spatial, and then multiplies the resulting feature map with the input feature map to perform adaptive feature refinement. The operating principle of CBAM on feature maps is shown in Fig. 2.

**Figure 3:**
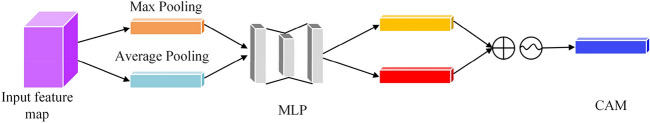
CAM module.

In the Channel Attention Module (CAM), the input feature map is processed, as shown in Fig. 3. First, the feature map passes through a parallel maximum pooling layer and an average pooling layer for feature recognition. Then it passes through the multi-layer perceptron module to process the output results. After adding elements one by one, the weight coefficient of the channel is obtained by a sigmoid activation function, and multiplied by the input feature map to obtain the weighted feature map.

**Figure 4:**
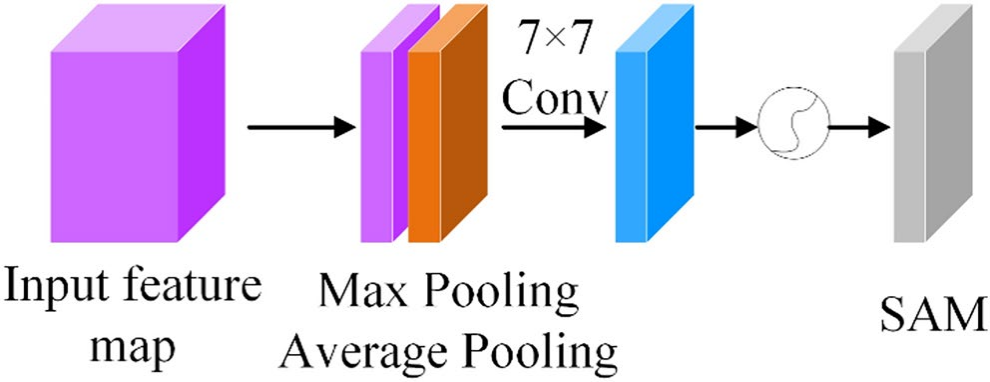
The structure of SAM Module.

In the Spatial Attention Module (SAM), the feature map is processed, as shown in Fig. 4. The input feature maps are processed by serial maximum pooling and average pooling respectively to generate two new feature maps. Then, these two feature maps are spliced by Concat operation, and after a 7 × 7 convolution, a single-channel feature map is obtained. Secondly, the feature map is generated by the sigmoid activation function to generate the spatial weight coefficient. Finally, the weighted feature map is obtained by multiplying the weight with the initial feature map.

**Figure 5:**
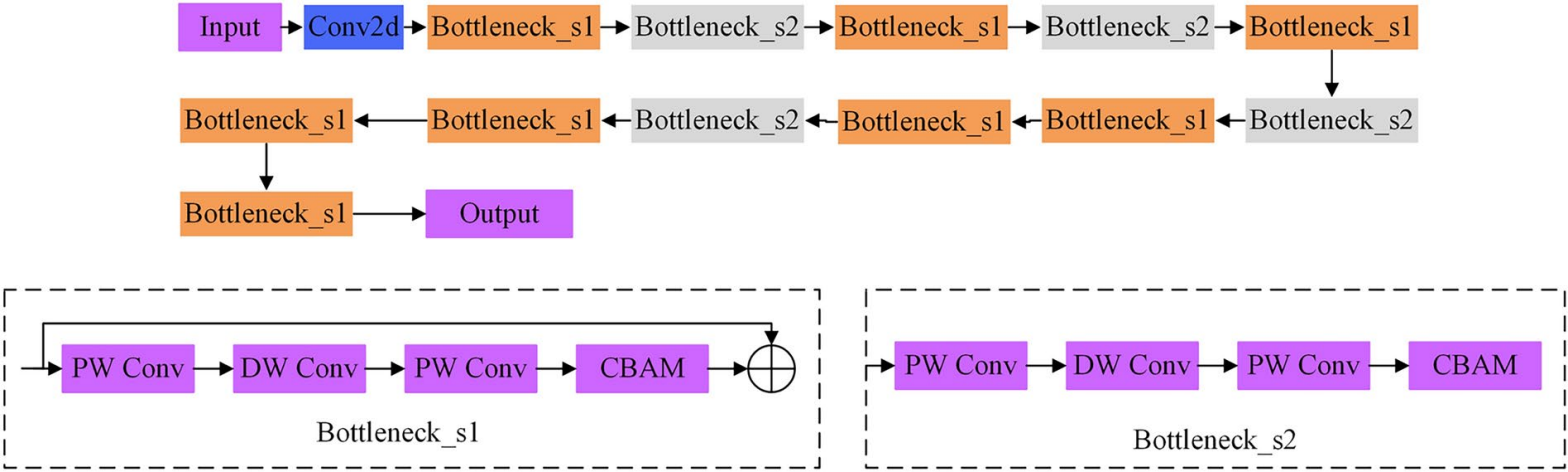
Improved MobileNet V2 network.

*Improved MobileNet V2 network* Aiming at the problem of extracting burned area from remote sensing images, this paper improves the MobileNet V2 model and designs an efficient burned area extraction algorithm. The structure of the improved MobileNet V2 model proposed in this article is shown in Fig. 5. It consists of input layer, ordinary convolution layer, PW convolution, DW convolution, CBAM, Bottleneck_1, Bottleneck_2 and pooling layer. The design idea of this article is to enhance the model’s ability to extract features of burned areas. Embed the attention mechanism in Bottleneck_1 and Bottleneck_2 to construct a basic unit with an attention mechanism. The CBAM attention mechanism can adaptively obtain the channel and spatial weight coefficients of the feature map. According to the corresponding weight coefficients, key feature information useful for burned area can be selected from complex feature information. Strengthening key feature information can effectively improve the model’s anti-noise performance and feature extraction capabilities. This study embeds the CBAM attention mechanism into the basic unit modules Bottleneck_1 and Bottleneck_2 with a step size of 1 and a step size of 2 in MobileNet V2, respectively. This enables the model to pay more attention to useful feature information and suppress useless feature information in both the channel dimension and the spatial dimension.

*Improved Deeplab V3* + *segmentation network* Deeplab V3 + was proposed by Google in 2018^35^. It is a typical encoding–decoding structure and is one of the most excellent semantic segmentation algorithms at this stage. This article improves on the original Deeplab V3 + network and uses the improved MobileNet V2 network to replace the original backbone network Xception. The network consists of two parts: encoder and decoder. The encoder is divided into improved MobileNet V2 network and Atrous Spatial Pyramid Pooling (ASPP). The decoder fuses low-level and high-level features and performs feature map recovery, as detailed below.

**Figure 6:**
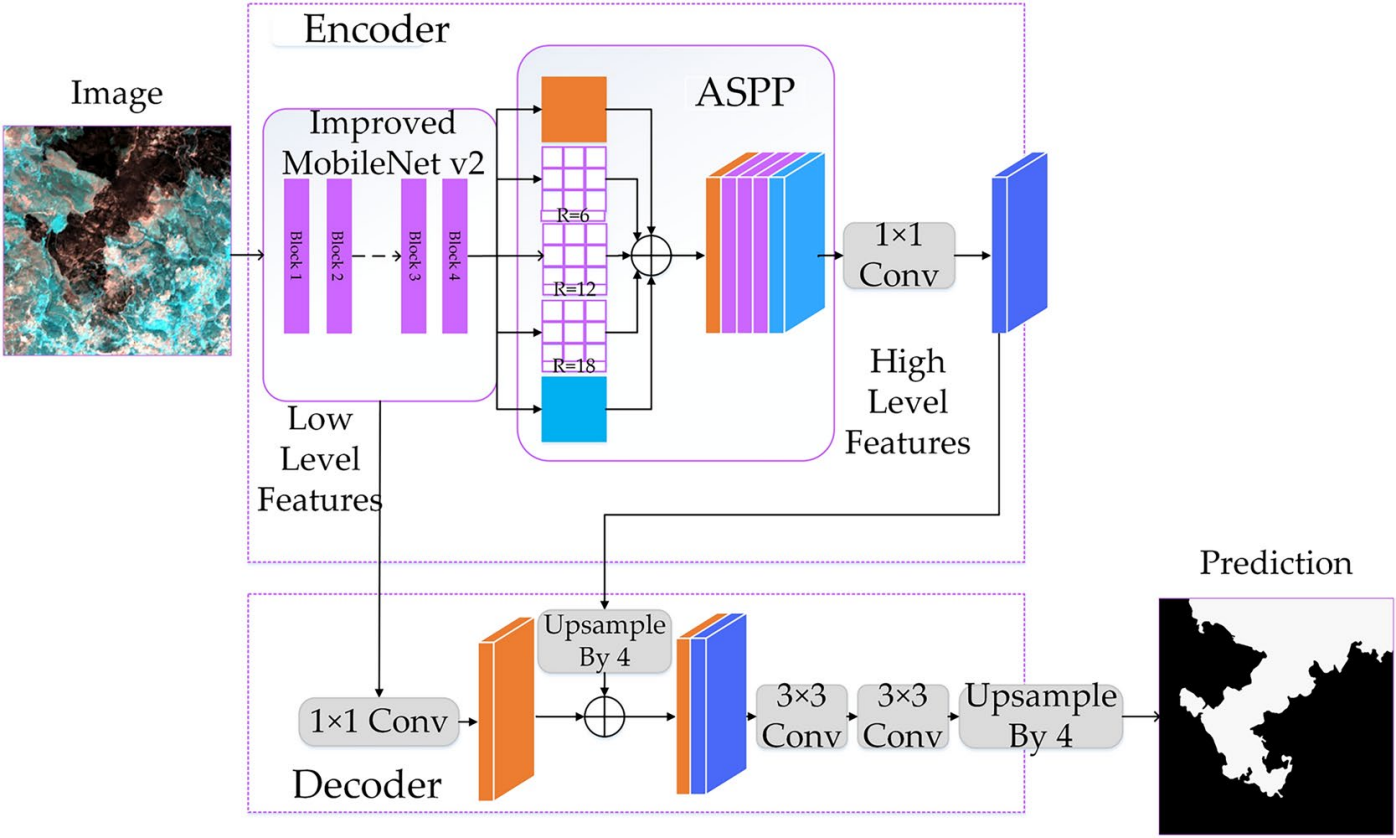
Improved Deeplab V3 + network.

First, the improved MobileNet V2 extracts features from burned area in remote sensing images to obtain low-level feature information. Since this article has made improvements in the MobileNet V2 network, compared with the original MobileNet V2 network, the features of burned area extracted are more accurate, and the improved parameters are smaller. It not only improves the accuracy of feature information of burned area but also rarely increases the number of parameters. Secondly, the low-level feature information is transferred to the decoding layer and ASPP module respectively. DeepLab V3 + network draws on the Spatial Pyramid Pooling (SPP) operation in SSP-Net^20,36^ and improves it into ASPP to further improve the accuracy of classification. In ASPP, the input low-level burned area feature maps are subjected to 1 × 1 convolution, 3 × 3 convolution with expansion rates of 6, 12 and 18, and global average pooling operations, and then the feature maps are fused and 1 × 1 convolution. Obtain high-level burned area feature information. Finally, in order to fully extract high-level feature information of burned area, this paper performs necessary down-sampling operations on the input image. In order to make up for the boundary information lost in the down-sampling operation, the network in this paper fuses low-level features during the feature map recovery process to restore the boundary information of the target part. The linear interpolation method is used to restore the feature map. Finally, the accuracy of network segmentation is improved, and a meaningful burned area segmentation map is obtained (Fig. 6).

**Figure 7:**
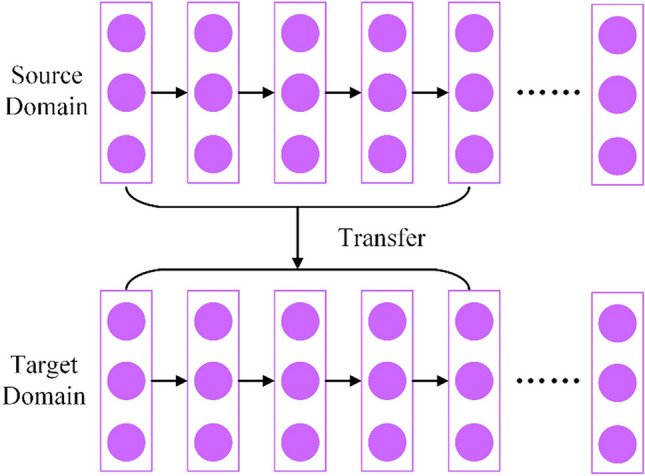
Transfer learning principle.

*Extraction strategy for burned areas* In remote sensing image target extraction based on deep neural networks, the phenomenon of big data and few annotations is common. This phenomenon results in the inability to provide rich feature information of the network, resulting in generally low extraction accuracy. Transfer learning is a good idea, which can apply knowledge learned from one environment to another new learning task. That is, from the given source domain Ds = {Xs, Fs(X)} and learning task Ts, migrate to the target domain Dt = {Xt, Ft(X)} and its learning task Tt. Transfer learning can be divided into four categories, namely instance-based deep transfer learning, mapping-based deep transfer learning, network-based deep transfer learning and adversarial-based deep transfer learning^37^. This article applies network-based deep transfer learning. As shown in Fig. 7, network-based deep transfer learning applies the network structure and connection parameters trained in the source domain to the network structure of the target domain and becomes part of the target domain network structure. Finally, the purpose of improving extraction accuracy is achieved by fine-tuning the target domain network.

**Figure 8:**
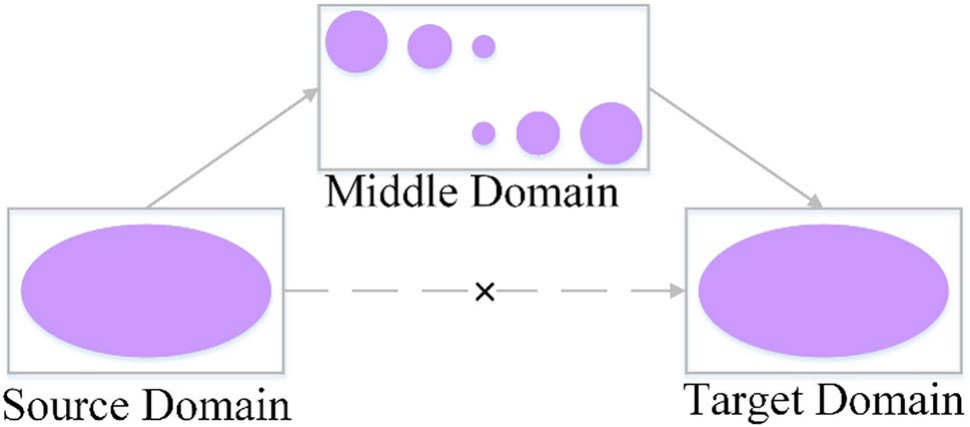
Transitive transfer learning.

Transfer learning can alleviate the problem of accuracy decline caused by insufficient deep learning sample size to a certain extent. The common method is fine-tuning. Fine-tuning is to adjust the structure of the pre-trained network model and use it in the network training of this article. The advantage of this is that it can improve training efficiency and achieve good training results on small-scale data sets. However, the disadvantage is that the characteristics of the source domain and the target domain are quite different, and the improvement effect of transfer learning will be reduced. The ImageNet is a dataset widely used to train deep learning models. It contains a total of 1.4 million images and has rich information features. ImageNet pre-trained weights are commonly used for transfer learning through fine-tuning to alleviate the problem of insufficient samples, thereby improving classification accuracy^38^. verified through multiple experiments that whether migrating on similar data sets or dissimilar data sets. The effect of using migration weight initialization will be higher than random initialization to extract the target. Although the network model pre-trained on the ImageNet dataset has rich underlying features, there is a large gap between the image features of the IMageNet dataset and remote sensing images, and the improvement in classification effect is limited. Transitive transfer Learning (TTL) was proposed by Professor Yang’s team in 2015^39^. Its purpose is to solve the problem of large gaps between the data in the source domain and the characteristics of the target domain. The core idea of transitive transfer learning is that if the gap between the source domain data set and the target domain data set is large, one or more intermediate domain data sets with certain similarity to both are introduced to link them, and the rich underlying features of the source domain can be utilized. The principle is shown in Fig. 8.

**Figure 9:**
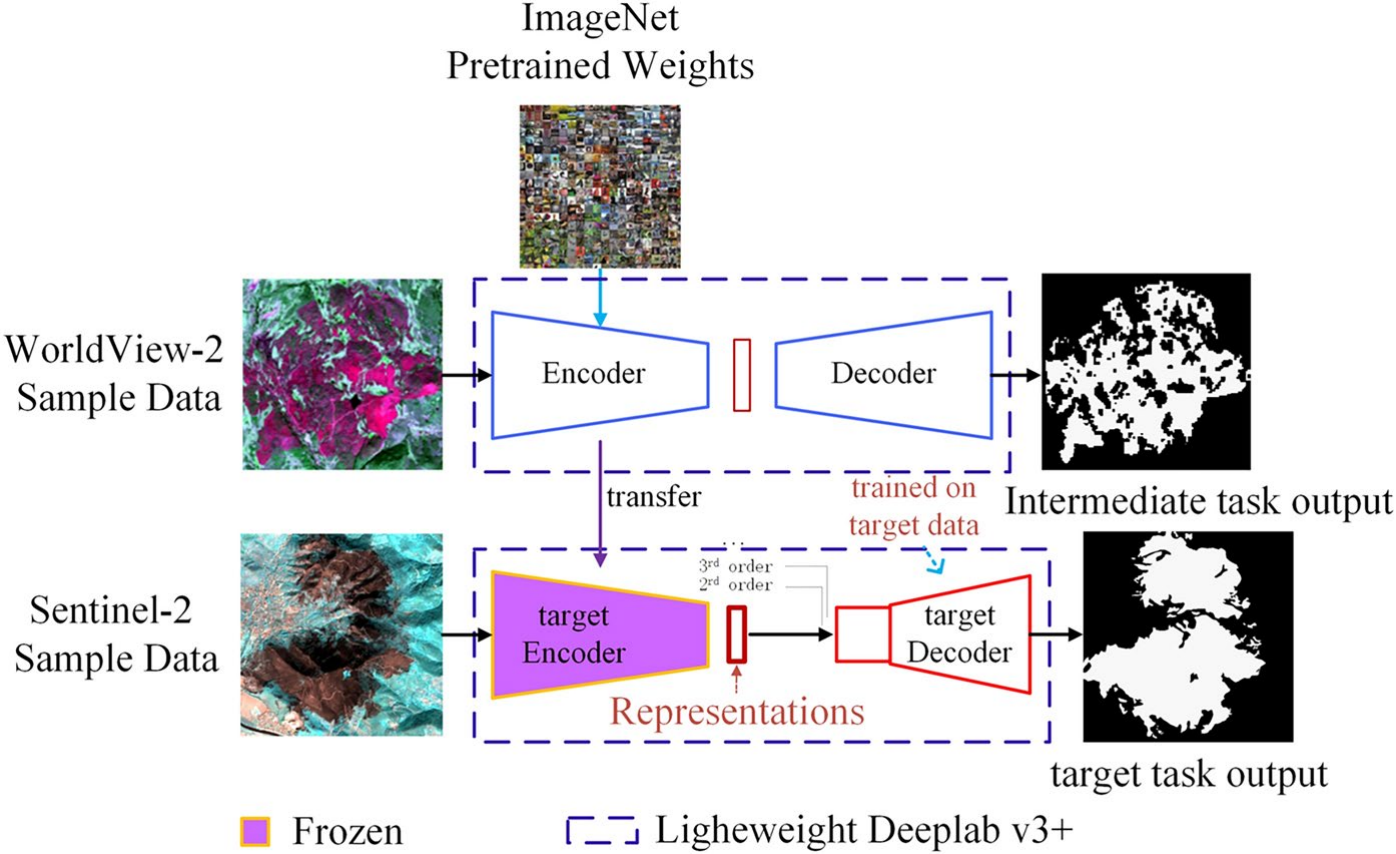
The network training process of this article.

The deep transitive transfer learning used in this article is to apply the pre-trained weights in the ImageNet data set to the burned area extraction task of the WorldView-2 sample data set. Then the feature parameters trained on the WorldView-2 sample data set are applied to the burned area extraction task of the Sentinel-2 data set. First, rich underlying features are extracted through the improved backbone network MobileNet V2, and through fine-tuning, we have richer deep features and complete the migration from the ImageNet data set to the WorldView-2 data set. Then, the improved Deeplab V3 + network is used as the classifier, and the network weights trained by the WorldView-2 data set are transferred to the network training of the Sentinel-2 data set. In the training of the Sentinel-2 data set, the encoding layer of the improved Deeplab V3 + network is first frozen, and only the decoding layer part is trained. This is to prevent over-fitting caused by too few training samples, and at the same time save training time to achieve the purpose of short time consumption. Then, the encoding layer part is unfrozen, and the Sentinel-2 dataset extracts the underlying features in the encoding layer part and fuses them with the underlying features extracted from the WorldView-2 dataset. Finally, the migration of the WorldView-2 data set to the Sentinel-2 data set is completed through fine-tuning. It is more direct to refer to the pre-trained weights of the ImageNet data set on the Sentinel-2 data set, and under the same number of iterations, it has higher burned area extraction accuracy. At this point, the migration from the ImageNet data set to the WorldView-2 data set and finally to the Sentinel-2 data set is completed (Fig. 9).

## Results

*Target domain dataset* The data used in this article is divided into two parts. The first part is the target domain data set, which is used to train the improved Deeplab V3 + network. The types of features in the image are divided into two categories: burned area and other features. The image data comes from eight countries including Portugal, Greece and Spain, and was obtained from July and August from 2019 to 2023. The image is a multispectral image acquired by Sentinel-2, with a ground pixel resolution of 10 m. Its four bands of red, green, blue and near-infrared were selected to synthesize a true-color image as the experimental image data of this article. Table 1 shows partial image and label information. In this experiment, the original image was made into a label map through ArcGIS 10.2 software. The size was cropped into a sample image of 512 × 512 pixels, and samples without burned area features were removed, resulting in a total of 136 sample images. Finally, the data set was expanded by using rotations of 90°, 180°, 270°, vertical mirroring, and horizontal mirroring, and a total of 680 labeled samples were obtained. Among them, 90% were randomly selected as training samples, and 10% were used as test samples and verification samples.

**Table 1:** Target and dataset information.

Image	Label	Area	Time	Coordinate
		Ortugal	2023–08-06	7° 47′ 27.137″ W-39° 45 ′23.796″ N
		Greece	2023–08-28	23° 39′ 10.221″ E-38° 7′7.989″ N
		Spain	2022–08-19	20° 42′ 16.911″ W-39° 53′ 26.063″ N

**Figure 10:**
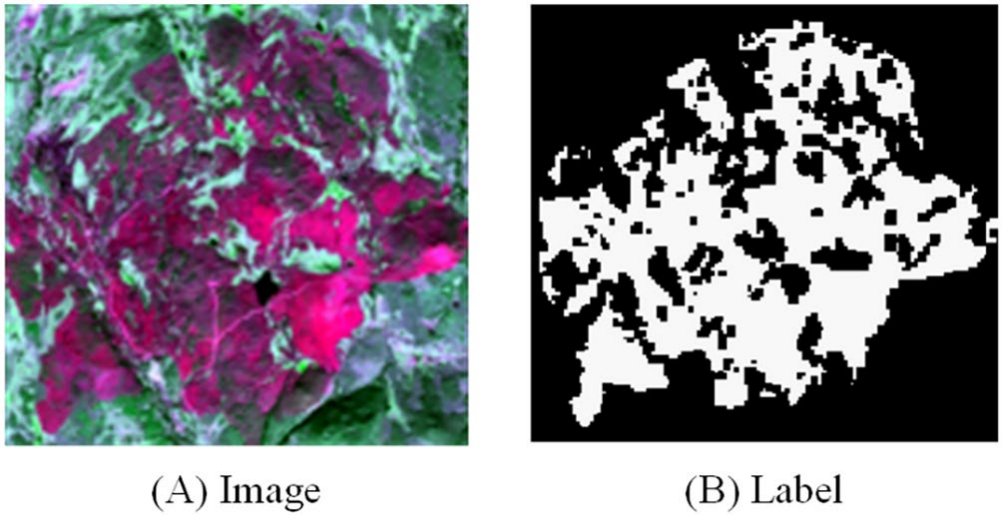
WorldView-2 image and label.

*Intermediate domain dataset* The second part is the intermediate domain dataset. WorldView-2 data downloaded at the Copernicus Emergency Management Service (EMS) serves as an intermediate domain dataset corresponding to training and test images (https: //emergency. copernicus. eu/ mapping/ list-of-activations). This product has been used in previous studies as reference data for burned area detection and burn severity estimation. The resolution is 2.0 m, and the cloud coverage is approximately 0%. This article selects some of the images and crops them to obtain 280 images with a pixel size of 512 × 512 pixels. Finally, the data set was expanded by using rotations of 90°, 180°, 270°, vertical mirroring, and horizontal mirroring, and a total of 1,400 labeled samples were obtained. The experimental dataset, test dataset, and validation dataset are partitioned consistently with the target domain (Fig. 10).

*Environmental configuration and accuracy evaluation indicators* The experimental environment of this article is the Windows 10 operating system, and the experimental network in this paper is trained on the high-level neural network application program interface Keras 2.2.4 based on Tensorflow1.13.1. In terms of hardware, the processor is an Intel(R) Xeon(R) CPUE5-4650 graphics workstation and equipped with an NVIDIA Tesla K40c GPU computing card. In terms of training parameters, adaptive moment estimation (Adam)^40^ is used, epoch is set to 100, and batch size is set to 4. SegNet, U-Net and the original Deeplab V3 + were selected as comparison algorithms. And the Intersection-over-Union ratio (MIoU), Kappa coefficient, and Overall Accuracy (OA) were selected as accuracy evaluation indicators.The complexity evaluation indicators are model Parameters (Params), Floating Point Operations (FLOPs) and training-time, where FLOPs represents the amount of Floating Point Operations in the forward inference process of the model. The calculation formulas of MIoU, OA and Kappa coefficient are respectively.

1 $$\text{MIoU=TP/(TP+FN+FP)}$$

2 $$\text{OA=(TP+TN)/(TP+FN+FP+TN)}$$

3 $$\text{Kappa=(P0-}\text{Pe)/(1-Pe )}$$

where TP is the number of positive samples classified as burned areas by the model. FN is the number of positive samples classified as other land types by the model. The number of negative samples in which FP was mistakenly classified as other land types by the model; the number of negative samples in which TN was classified as burned area land by the model. Po is the sum of the number of correctly classified samples of each category divided by the total number of samples; Pe is the sum of the product of the number of real samples and the number of predicted samples of each category divided by the square of the total number of samples.

**Figure 11:**
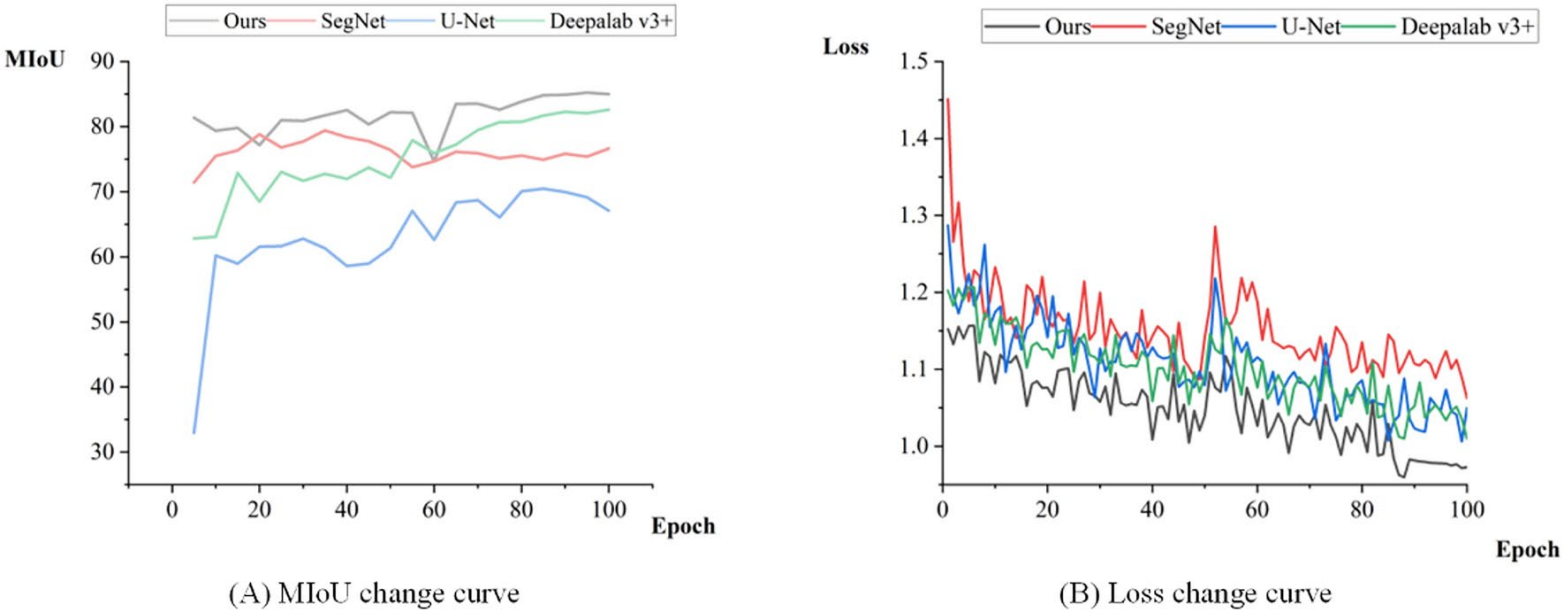
Training curve.

*Training* This article uses the improved Deeplab V3 + network model to train the fire-scarred land data set. The training was done. In order to test the effectiveness of the proposed algorithm and take into account the performance of the experimental equipment, the batch_size of each experiment was set to 4. The initial learning rate was set to 0.0001, and the epoch was set to 100.The training results are recorded every 5 epochs during training, and MIoU is calculated to test the segmentation accuracy. Figure 11(a) shows the changes in MIoU during the training period. The segmentation accuracy of the model increases with the increase in the number of training iterations. When Epoch reaches more than 70 times, the MIoU of each algorithm is basically stable, with fluctuations of less than two percentage points. The results show that this method has higher segmentation accuracy and stronger robustness than other methods. Figure 11(b) shows the value of Loss during the training period. As Epoch increases, the Loss curve continues to converge. When Epoch reaches close to 90 times, the curves of each model are relatively flat, indicating that each model has reached a stable state.

**Figure 12:**
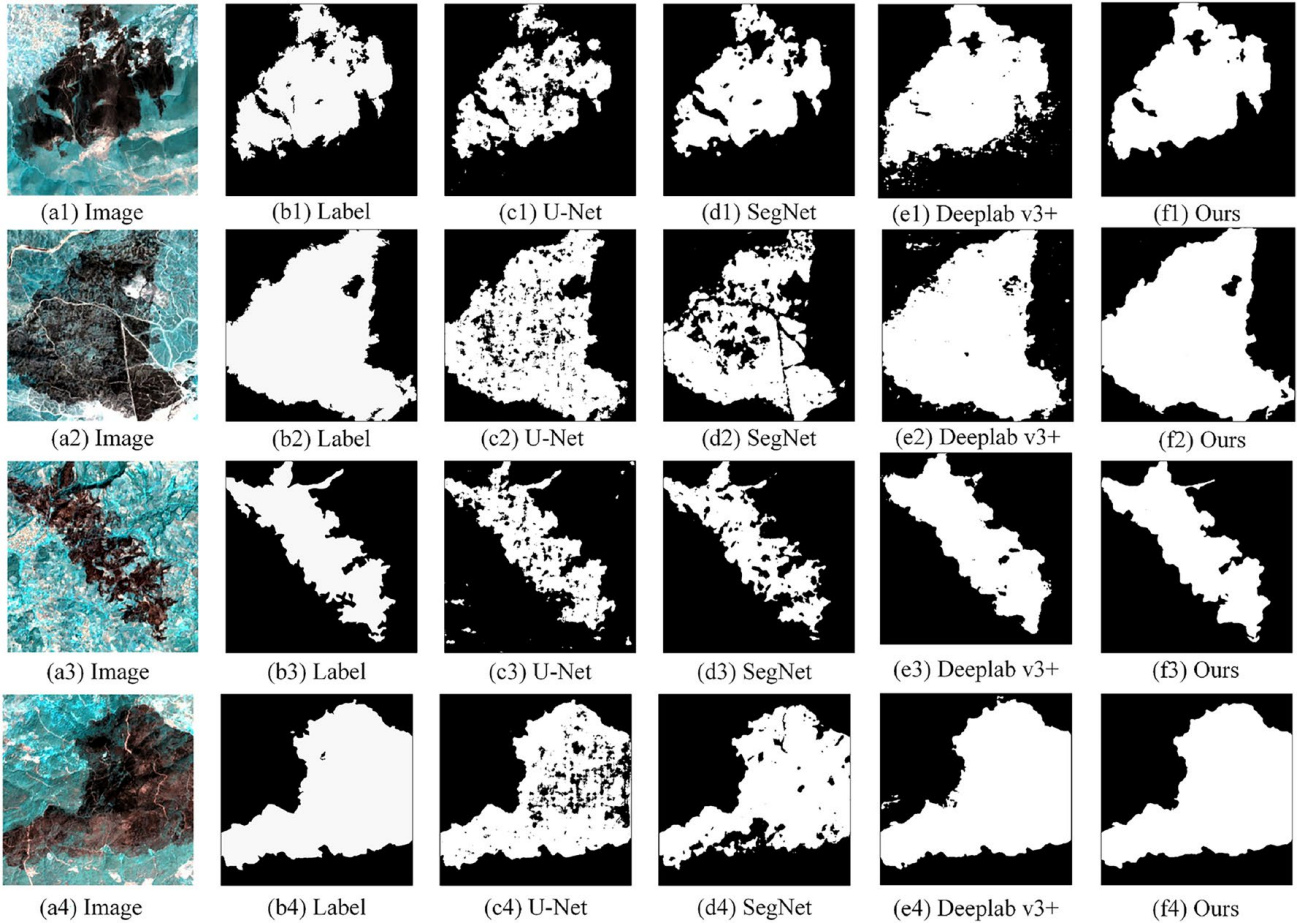
Comparative experimental extraction results.

*Comparative experiment* In order to verify the effectiveness and superiority of this method, this article conducts experiments using SegNet, U-Net, Deeplab V3 + and the proposed method based on deep transfer learning proposed in this article, and compares and analyzes the experimental results. Some experimental results are shown in Fig. 12. Figure 12a–f respectively show the image, label, U-Net extraction results, SegNet extraction results, Deeplab V3 + extraction results and the extraction results of this method.

As can be seen from Fig. 12, in the four groups of experiments, the extraction effect of U-Net is poor, the extraction results have serious holes, and the integrity is poor. Moreover, there is still an inaccurate extraction phenomenon in Fig. 12c3. The extraction results of SegNet are more complete than those of U-Net, and the hole phenomenon is weakened, but the continuity of the extraction results is still poor. The extraction results of Deeplab v3 + are due to U-Net and SegNet. The hole phenomenon is significantly improved, and the continuity of the extraction results is better. However, the edge detail processing of the extraction results of Deeplab v3 + is still unsatisfactory. The extraction results may incorrectly extract other small features. The extraction results of this method have been significantly improved in the hole phenomenon, and the extraction results have better continuity. This is due to the CBAM module cited in this article, which improves the screening of feature information of burned area and can accurately identify burned area. In addition, the deep transfer learning strategy introduced in this article has more completely supplemented the feature information of the burned area, making the edge details of the extraction results more detailed and the overall extraction results more complete. All of the above show that the method in this paper is more effective and superior and can be applied to the task of extracting burned area.

Table 2 shows the quantitative evaluation results of burned area extraction using the comparative method. It can be seen that the MIoU of the U-Net network and SegNet network are 62.30% and 70.77%, respectively, which are both lower than the Deeplab V3 + network. The method in this article is 10.12% higher than Deeplab V3 + in MIoU. In terms of OA, this method exceeds 90%, while other methods are lower than 90%. The Kappa coefficient of the U-Net and SegNet network are 0.5171 and 0.6534, respectively. The Deeplab V3 + network is 0.6921, and the Kappa of this method is 0.8190, which is much higher than the other three methods. The above quantitative indicators illustrate that the method in this article uses the CBAM module and deep transitive transfer learning, which has improved the recognition of burned areas and can effectively extract burned area. Using the improved MobileNet V2 as the backbone network, Params and FLOPs are significantly better than DeepLab V3 + , which shows that this method saves a lot of parameter calculations. In terms of training time, it saves 4.16h compared to U-Net and 13.36 h compared to Deeplab V3 + . It can be seen from the above indicators that the algorithm in this paper has improved compared with other methods in extracting burned area, and has also fully improved in training time, which illustrates the feasibility of this algorithm.

**Table 2:** Comparative experimental accuracy evaluation.

Method	MIoU (%)	OA (%)	Kappa	Params (MB)	FLOPs (GB)	Training-time (h)
U-Net	62.30	80.55	0.5174	7.78	34.60	23.22
SegNet	70.77	84.48	0.6534	30.53	80.40	27.06
Deeplab V3 +	73.54	86.36	0.6921	41.53	105.63	32.42
Ours	83.62	92.97	0.8190	2.83	13.56	19.06

*Ablation experiment* In order to verify the effectiveness and necessity of each module of the model in this article, experiments were conducted on the verification set using the method in this article and its variants. The Deeplab V3 + network is the original Deeplab V3 + network with Xception as the backbone network. LDeeplab V3 + network is a Deeplab V3 + network with MobileNet V2 as the backbone network. The CB-Deeplab V3 + network is a Deeplab V3 + network with the improved MobileNet v2 network as the backbone network. The TCB-Deeplab V3 + network is a Deeplab V3 + network that directly migrates ImageNet to the Sentinel-2 dataset and uses the improved MobileNet v2 network as the backbone network. The network in this article is the Deeplab V3 + network that applies deep transitive transfer learning and improved MobileNet. As shown in Table 3.

**Table 3:** Ablation experiment analysis table.

Method	Xception	MobileNet V2	MobileNet V2 + CBAM	TL	DTTL
Deeplab v3 +	√				
LDeeplab v3 +		√			
CB-Deeplab v3 +			√		
TCB-Deeplab v3 +			√	√	
Ours			√		√

**Figure 13:**
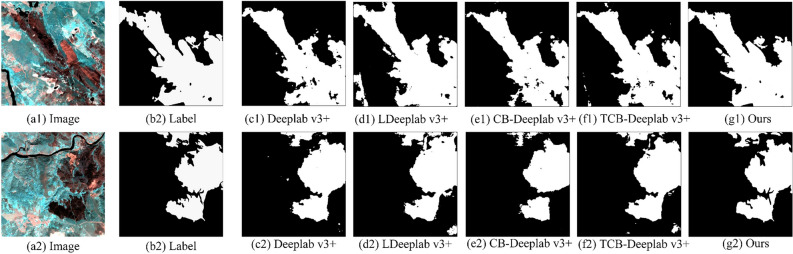
Extraction results of ablation experiment.

As can be seen from Fig. 13, there are holes in the extraction results of Deeplab V3 + , the edge details are poorly processed, and some burned areas are not fully extracted. The holes in the extraction results of LDeeplab V3 + disappear, but the extraction is inaccurate. The CB-Deeplab v3 + network quotes CBAM on the basis of the LDeeplab V3 + network, which further enhances the identification ability. The error extraction situation has been significantly improved, and edge details have also been improved. However, overall there are still problems such as incomplete extraction. TCB-Deeplab v3 + refers to transfer learning in CB-Deeplab v3 + . It can be seen from the results that the extraction results have been improved, the extraction of edge detail information has been improved, and the identification of burned areas has been strengthened. The method in this article uses the deep transitive transfer learning strategy based on the CB-Deeplab V3 + network. By citing the underlying information of the ImageNet data set and the feature information of burned area in the intermediate domain of WorldView-2, this article’s ability to identify burned area is further enhanced. Therefore, compared with the previous methods, the extraction results of this method are more accurate and continuous, and the edge details are more complete. The above illustrates the effectiveness and necessity of using CBAM and deep transitive transfer learning in this method.

It can be seen from Table 4 that LDeeplab V3 + uses MobileNet V2 as the backbone network. Compared with the original Deeplab V3 + network, LDeeplab V3 + is slightly lower than the original Deeplab V3 + network in MIoU, OA and Kappa, but it has great advantages in Params, FLOPs and Training-time. When the improved MobileNet V2 network is used as the backbone network, MIoU, OA and Kappa are improved, increasing by 7.15%, 2.71% and 0.0941 respectively. The network in this article uses DTTL based on the CB-Deeplab v3 + network, and all indicators reach the maximum. There is a small increase in Params, FLOPs and Training-time. The above indicators illustrate that the method in this paper is effective and robust in improvement.

**Table 4:** Accuracy evaluation of ablation experiments.

Method	MIoU (%)	OA (%)	Kappa	Params (MB)	FLOPs (GB)	Training-time (h)
Deeplab v3 +	73.54	86.36	0.6921	41.53	105.63	32.42
LDeeplab v3 +	72.97	85.93	0.6841	2.77	11.517	18.99
CB-Deeplab v3 +	80.12	88.64	0.7782	2.85	13.778	19.06
TCB-Deeplab v3 +	80.75	89.54	0.7835	2.85	13.778	19.06
Ours	83.62	92.97	0.8190	2.85	13.778	19.06

**Figure 14:**
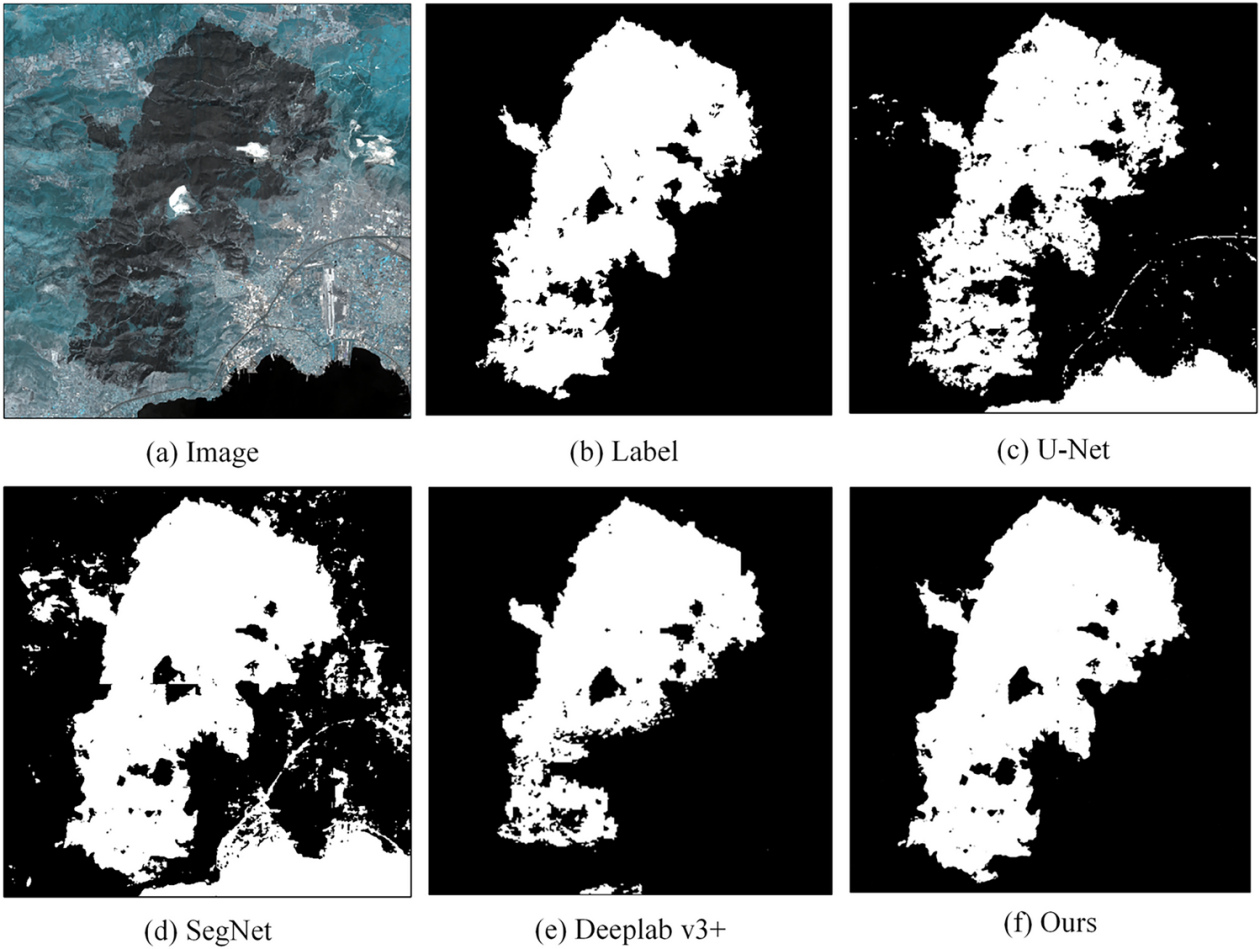
Western Attica burned area extraction results.

*Extraction of burned area from large-scale remote sensing images* In order to verify the applicability and applicability of this method in large-scale remote sensing images, this method was applied to the Western Attica region of Greece to extract burned area. The Western Attica is a fire-prone area. The remote sensing image was captured on July 20, 2023. During the imaging process, due to the sensor itself and the absorption and scattering of the atmosphere during the propagation of electromagnetic waves. There is a difference between the gray value of the image and the actual radiance value of the ground. Therefore, this paper performed a series of preprocessing operations on the data, including radiometric calibration, atmospheric correction, image enhancement, image cropping, and image splicing, to obtain complete remote sensing image data of the study area. Figure 14a-f below show the remote sensing images, labels, U-Net extraction results, SegNet extraction results, Deeplab V3 + extraction results and the extraction results of this method respectively.

Apply U-Net, SegNet, Deeplab V3 + and the algorithm of this article to extract burned area in the Western Attica region of Greece. The inference time of each algorithm is 0.23 h, 0.34 h, 0.46 h and 0.12 h respectively. The algorithm in this paper has a great advantage in the inference time of large-scale remote sensing images, saving 0.34 h compared with the original Deeplab V3 + network. As can be seen in Fig. 14c, the burned area extraction results of U-Net are seriously fragmented, have poor integrity and have erroneous extractions. In Fig. 14d, SegNet extraction results are better in terms of completeness than U-Net, but the problem of inaccurate extraction still exists. In Fig. 14e, the extraction results of Deeplab V3 + are better than the first two methods, and the problem of wrong extraction is significantly improved, but the edge detail processing is poor. As can be seen from Fig. 14f, the algorithm in this paper has achieved good results in extracting burned area, showing good performance in terms of completeness, and the problem of incorrect extraction has been significantly improved. As can be seen from Table 5, when extracting large-scale remote sensing images, the accuracy of SegNet and U-Net networks decreases, while the Deeplab V3 + network and the method in this paper achieve better performance. The non-uniqueness of the burned area is a difficult problem to extract, but the algorithm in this paper can effectively identify it using the CBAM module. The detailed information has been further enhanced under the action of deep transfer learning. This makes the burned area extraction results of this method relatively complete and the edge details processed better. The above shows that the algorithm in this paper has high application value in extracting large-scale burned area. In forest security protection, accurate and rapid burned area extraction provides data support for this work and saves a lot of manpower and material resources. In addition, the algorithm in this article will also provide certain help and support for disaster prevention and environmental monitoring in forest areas.

**Table 5:** Large-scale experimental accuracy verification.

Method	MIoU (%)	OA (%)	Kappa
U-Net	60.20	78.22	0.5012
SegNet	59.36	76.20	0.4915
Deeplab V3 +	75.54	87.23	0.7021
Ours	83.84	93.58	0.8265

## Conclusion

This article proposes an improved Deeplab V3 + network suitable for forest burned area extraction. Based on the MobileNet V2 network, the CBAM module is integrated to add weight information in the dual dimensions of channel and space of the feature map. This can effectively inhibit the network from learning non-burned area information, making the proposed network more efficient and targeted. The improved lightweight network MobileNet V2 is used to replace the Xception network in the original Deeplab V3 + network as the main framework. While improving the accuracy of burned area extraction, the training speed is improved by reducing the number of parameters. Introducing deep transitive transfer learning, the method in this paper can further enhance the recognition ability of feature information of burned area and improve the extraction accuracy. Using U-Net, SegNet, Deeplab V3 + to compare with the algorithm in this article. The results show that according to the characteristics of the burned area, the algorithm proposed in this paper can accurately identify the burned area and ensure the continuity and edge details of the burned area. Quantitative analysis of the burned area extraction results shows that the algorithm in this paper is better than the other three comparison methods in various evaluation indicators. Among them, OA exceeds 92% and Kappa exceeds 0.81. And the training speed is faster. In the ablation experiment, the effectiveness and necessity of the improvement were verified in the experiment. In summary, it shows that the algorithm in this paper is more efficient, accurate and targeted. The algorithm in this article was applied to the Western Attica region of Greece to extract burned area, and achieved good application results. The algorithm in this article has made some breakthroughs in the task of extracting burned area. However, as the resolution of remote sensing image data continues to improve and the amount of data continues to increase, the time to extract burn area will also increase. Regarding future work, the following two points may be promising or deserve special attention.

The advantages and disadvantages of this method are summarized as follows. (1) MobileNet V2: MobileNet V2 serves as the foundational network architecture in the proposed approach. It is known for its lightweight design, making it suitable for resource-constrained environments such as mobile devices or edge computing. The advantage of using MobileNet V2 lies in its ability to provide a good balance between model size and performance. However, its shallow depth compared to some other networks might limit its capacity to capture intricate features, which could potentially affect the accuracy of the extracted burned areas. (2) CBAM Module: The inclusion of the CBAM is a significant enhancement to the network. This module helps the network focus on relevant features by weighting information in both channel and spatial dimensions of the feature map. This attention mechanism aids in suppressing irrelevant information, thereby improving the efficiency and specificity of the network. The advantage here is that it helps mitigate the risk of learning non-burned area information, enhancing the overall accuracy of burned area extraction. However, the drawback could be the additional computational cost incurred by integrating this module, which might impact inference speed, especially on resource-constrained devices. (3) Deep Transitive Transfer Learning: The method employs deep transitive transfer learning to further boost the recognition capability of burned area feature information. Transfer learning leverages knowledge gained from one task or domain to improve learning and performance in another. By adapting pre-trained models to the specific task of burned area extraction, the approach benefits from learned features that are relevant to the task. The advantage of transfer learning is its ability to expedite training and potentially improve performance, especially when labeled data for the target task is limited. However, the drawback lies in the assumption that features learned from the source task are transferable and beneficial for the target task. If the source and target tasks differ significantly, transfer learning might not yield the expected performance improvements. (4) Performance Evaluation: The approach is rigorously evaluated against established algorithms such as U-Net, SegNet, and Deeplab V3 + . The quantitative analysis demonstrates superior performance in accurately identifying burned areas while preserving continuity and edge details. The advantage here is the thorough comparison with state-of-the-art methods, providing a benchmark for assessing the efficacy of the proposed approach. However, the drawback could be the limited scope of comparison, as there may exist other algorithms or variations that were not included in the evaluation.

## Supplementary Information

Supplementary Information 1.

Supplementary Information 2.

## Data Availability

The data presented in the study are available on request from the first and corresponding author. The data are not publicly available due to the thesis that is being prepared from these data.
